# Assessing Transparency of Robots, Exoskeletons, and Assistive Devices: A Systematic Review

**DOI:** 10.3390/s25144444

**Published:** 2025-07-17

**Authors:** Nicol Moscatelli, Cristina Brambilla, Valentina Lanzani, Lorenzo Molinari Tosatti, Alessandro Scano

**Affiliations:** Institute of Intelligent Industrial Technologies and Systems for Advanced Manufacturing (STIIMA), Italian National Research Council (CNR), 20139 Milano, Italy; cristina.brambilla@stiima.cnr.it (C.B.); valentina.lanzani@stiima.cnr.it (V.L.); lorenzo.molinaritosatti@stiima.cnr.it (L.M.T.); alessandro.scano@stiima.cnr.it (A.S.)

**Keywords:** transparency, exoskeletons, assistive devices, rehabilitation, industry, motor control

## Abstract

Transparency is a key requirement for some classes of robots, exoskeletons, and assistive devices (READs), where safe and efficient human–robot interaction is crucial. Typical fields that require transparency are rehabilitation and industrial contexts. However, the definitions of transparency adopted in the literature are heterogeneous. It follows that there is a need to clarify, summarize, and assess how transparency is commonly defined and measured. Thus, the goal of this review is to systematically examine how transparency is conceptualized and evaluated across studies. To this end, we performed a structured search across three major scientific databases. After a thorough screening process, 20 out of 400 identified articles were further examined and included in this review. Despite being recognized as a desirable and essential characteristic of READs in many domains of application, our findings reveal that transparency is still inconsistently defined and evaluated, which limits comparability across studies and hinders the development of standardized evaluation frameworks. Indeed, our screening found significant heterogeneity in both terminology and evaluation methods. The majority of the studies used either a mechanical or a kinematic definition, mostly focusing on the intrinsic behavior of the device and frequently giving little attention to the device impact of the user and on the user’s perception. Furthermore, user-centered or physiological assessments could be examined further, since evaluation metrics are usually based on kinematic and robot mechanical metrics. Only a few studies have examined the underlying motor control strategies, using more in-depth methods such as muscle synergy analysis. These findings highlight the need for a shared taxonomy and a standardized framework for transparency evaluation. Such efforts would enable more reliable comparisons between studies and support the development of more effective and user-centered READs.

## 1. Introduction

### 1.1. Robots, Exoskeletons, and Assistive Devices

Over the past decades, robots, exoskeletons, and assistive devices, which will be referred to as READs, have gained increasing interest in a variety of fields, including industrial applications, daily-life support, medical rehabilitation, and sports [1,2,3]. End-effector robotic devices such as manipulandum-type systems are programmable, automated machines capable of interacting with the environment, guiding or assisting human motion [4]. They establish contact only at the distal portion of the body, such as the hand, as happens in upper-limb applications [5,6]. In contrast, exoskeletons are multi-contact, wearable robotic devices designed to provide assistance or support to one or multiple body segments during the execution of specific tasks [7,8]. Lastly, assistive technologies, such as orthoses, are typically simpler tools designed to support individuals with physical or cognitive impairments.

READs can be divided into different categories depending on the body segment they support (upper limb, lower limb, or full body) or the type of intervention they provide (active or passive). In particular, active devices are equipped with one or more actuators that can either augment human power or assist in generating movement. Conversely, passive systems offer postural support or motion assistance by leveraging their intrinsic mechanical structures, without using any actuators. For instance, passive exoskeletons can enhance posture and reduce physical strain by storing energy during prolonged or heavy lifting tasks [9].

### 1.2. Applications

READs have been employed in many applications, especially in industrial, medical, and daily-life scenarios.

#### 1.2.1. Industrial Applications

Musculoskeletal disorders (MSDs) are the most common work-related health issues in Europe. It is estimated that nearly 24% of European workers experience back pain, while 22% suffer from muscular pain [10]. This problem persists despite the ongoing trend towards automation, since many workers are still involved in heavy material handling (30%), repetitive and strenuous movements (63%), and awkward body postures (46%) [11]. In this context, wearable robotic and assistive devices represent a promising solution, as they have the potential to assist workers in performing repetitive or physically demanding tasks more efficiently, thus reducing the risk of developing MSDs and increasing productivity in industrial settings [1,9]. Indeed, several studies have investigated the effects of passive and active devices on users’ biomechanical parameters, reporting positive outcomes in terms of reduction of muscular effort, discomfort, and heart rate [9,12,13,14].

#### 1.2.2. Medical and Daily-Life Scenarios

Neurological disorders often lead to motor impairments that significantly impact daily functioning. In this context, READs are already an established tool for supporting rehabilitation and improving therapy outcomes. Among the conditions that could benefit from the adoption of READs, stroke is among the most critical. Indeed, stroke is one of the leading causes of death and long-term disability worldwide [15], affecting approximately 1.1 million people in Europe annually [16], with recent predictions expecting a further increase in incidence in the upcoming years [17]. Although the majority of stroke patients survive the traumatic event, more than a third are left with a lifelong disability [18]. Besides stroke, other neurological conditions such as spinal cord injury (SCI) [19], Parkinson’s disease [20], and multiple sclerosis [21] also cause significant motor impairments on both upper and lower limbs. Although conventional rehabilitation treatments can produce partial or complete functional recovery [6,22], optimal and effective interventions remain essential to improve patient outcomes.

In this context, robot-based rehabilitation has emerged as a promising approach for promoting motor recovery, since it exploits the intrinsic neuroplasticity of the brain. Several studies have already demonstrated that robotic and assistive rehabilitation devices can lead to positive outcomes [23,24], comparable or slightly superior to those achieved with conventional therapy [16]. In fact, such technologies provide key advantages, such as the possibility of delivering high-intensity, repetitive, and goal-oriented training, which are crucial factors for promoting motor recovery in neurologically impaired patients [25,26,27]. Additionally, the interactive and engaging nature of robotic systems could enhance patient adherence and motivation, further improving rehabilitation efficacy [6].

Despite these advantages, the high cost of robotic rehabilitation systems limits their widespread adoption. In order to expand rehabilitation beyond clinical settings, there is increasing interest in developing more accessible solutions, such as portable or home-based devices. These technologies hold potential not only for medical purposes but also for daily-life assistance [28,29] and even for improving athletic performance in sports [30].

### 1.3. Transparency in READs

Transparency is commonly defined as the ability of a device to not interfere with or influence human motion and to be non-perceptible to the user when assistance is not provided [6,31]. A device achieves full transparency if the interaction forces between the READs and the user are equal to zero and the movements performed while wearing the device closely match those performed without it [32]. In reality, perfect transparency is unachievable, as human movements cannot be fully predicted. Nevertheless, approaching this ideal condition is highly desirable in many applications, as it enhances the quality of human–robot interaction and facilitates the implementation of task-specific control strategies. Transparent READs should be capable of compensating for their own weight, friction, and inertia. Therefore, transparency is considered a fundamental prerequisite for many industrial and especially medical applications, serving as a baseline for more advanced functions, such as arm weight compensation or adaptive control laws [33].

READs should assist or support without imposing unnatural movements or postures, promoting safe and effective human–device interaction. Consequently, the concept of transparency has emerged as a desirable feature of these technologies. However, transparency is compromised “on purpose” when assistance is provided, as the devices apply forces distributed along the assisted limbs and impose specific control strategies during rehabilitation therapy [6].

For this reason, achieving a balance between maintaining high transparency and delivering effective assistance or support is essential to ensure that the device is both functional and comfortable from a user-centered perspective [6]. Moreover, in rehabilitation applications, it has been reported that the level of assistance can significantly influence motor recovery, as it interferes with the restoration of neural pathways underlying movement. To address this, assistance strategies such as assist-as-needed [6] have been developed to provide support only when necessary, promoting active patient involvement. When the device is not transparent, as it is assisting motion, quantifying to what extent and on what variables transparency is not maintained is of primary importance for understanding the outcomes of human–robot interaction.

Transparency is typically assessed by comparing movement-related metrics recorded under natural conditions while interacting with the device. In the literature, several approaches have been proposed to improve transparency in READs. These include optimizing the mechanical design to improve the physical interaction with the user, for instance, by adding passive degrees of freedom that release hyperstatic constraints, integrating force sensors to minimize interaction forces and allow the device to better follow the user’s movements, and implementing control strategies that actively compensate for the device’s own dynamics.

However, these approaches are adopted by a limited number of devices, and the issue of transparency remains poorly investigated in the literature [33].

Following these considerations, a systematic and comprehensive analysis of transparency is required in order to deepen the current understanding of human–device interaction and to promote the development of standardized evaluation methods that can guide the design of more transparent and user-centered devices.

### 1.4. Aim

Following recent advancements and the growing interest in enhancing human–device interaction, this systematic review aims to comprehensively analyze the concept of transparency in READs. Specifically, we aimed to examine how transparency is currently defined and evaluated across the literature. By providing a structured overview of assessment methods and their application to READs, this work seeks to foster a clearer and more unified understanding of transparency. The review also highlights the need for consistent and multidimensional evaluation frameworks to support future developments in the field.

## 2. Materials and Methods

This systematic review aims to address the following research questions:

RQ1: What is transparency, and how can it be defined? Is transparency a desirable feature in robotic and assistive devices?

RQ2: What are the most commonly used quantitative metrics for transparency evaluation?

RQ3: Which application domains and anatomical regions are primarily considered in the transparency evaluation of robotic and assistive devices?

To conduct the literature research, we followed PRISMA (Preferred Reporting Items for Systematic Reviews and Meta-Analyses) international guidelines [34].

### 2.1. Literature Research Strategy

Studies performing a transparency evaluation of robotic or assistive devices were included in our research. The literature research was conducted on Scopus, Web of Science, and PubMed by using the following logical combination of keywords: “transparency”, “exoskeleton”, “robot”, and “assistive device”. The research was applied to the Title, Abstract, or Keywords fields and was restricted to articles published in English up to March 2025. The complete research query used was

(TRANSPARENCY) AND ((EXOSKELETON) OR (ROBOT) OR (ASSISTIVE DEVICE)).

For the purpose of this review, we chose to only include the term “transparency,” excluding more specific terms that are sometimes used as synonyms (e.g., backdrivability, compliance, etc.). This decision was made to capture studies addressing the concept of transparency from a broader and more general perspective, rather than one limited to a specific domain. In fact, terms used as synonyms, such as backdrivability, refer mainly to the mechanical properties of the device rather than to a multifaceted definition of transparency that includes several domains of evaluation.

### 2.2. Eligibility Criteria

After removing duplicates, all identified papers were subjected to a screening process to assess their eligibility. No automation tools were used in this process. To be included in this review, the studies had to meet all the following criteria:

1. The research query had to appear in the title, abstract, or keywords.
2. The study had to explicitly define and discuss transparency.
3. The study had to include a quantitative assessment of transparency.
4. The paper had to be a full article (at least 4 pages).
5. The article had to be in English.

The full text of the selected articles was reviewed to confirm their relevance. Additionally, the references of the included articles were examined to identify any additional relevant publications.

### 2.3. Data Extraction

The main outcomes that were extracted from the selected studies were (i) the used definitions of transparency and (ii) any quantitative or qualitative metrics employed for assessment of transparency. Additional variables collected were as follows: (a) aim of the study; (b) type of device tested; (c) application domain; and (d) anatomical segment considered.

The results of the included studies were summarized by describing and qualitatively comparing the main outcomes of interest, with the aim of providing a clear overview of the literature. No formal meta-analysis or quantitative synthesis was performed. The studies were grouped according to their characteristics, the definition of transparency, and the quantitative metrics they included. Moreover, tables and graphics were included to summarize and visually display the main results.

### 2.4. Risk of Bias Assessment

Given the conceptual nature of this review, no formal tool for risk of bias assessment was applied. Instead, we adopted a qualitative evaluation of potential sources of bias that could affect the selection and interpretation of the literature. For each identified bias, a specific mitigation strategy was applied to ensure a neutral and objective synthesis of the current literature.

To reduce any potential source selection bias, articles were selected based on clear inclusion criteria. Moreover, all valid, indexed articles that included the selected keywords were considered, regardless of their disciplinary background or the year in which they were published. Finally, the authors did not approach this review with a pre-existing thesis or expectation, but instead objectively organized and presented the current state of the art based on the available evidence.

### 2.5. Sensitivity Analysis

To evaluate the robustness of the conclusions, a qualitative sensitivity analysis was conducted considering only the most relevant studies and by checking whether the highest-quality studies would suggest the same outcomes as the full review. The highest-ranked studies were determined based on criteria such as the number of citations per year, the number of participants, and whether they included a systematic statistical analysis to confirm their findings. The results obtained from this subset were compared to the results of the entire set of studies to verify the consistency of the conclusions when only the top studies were included.

## 3. Results

**Figure 1:**
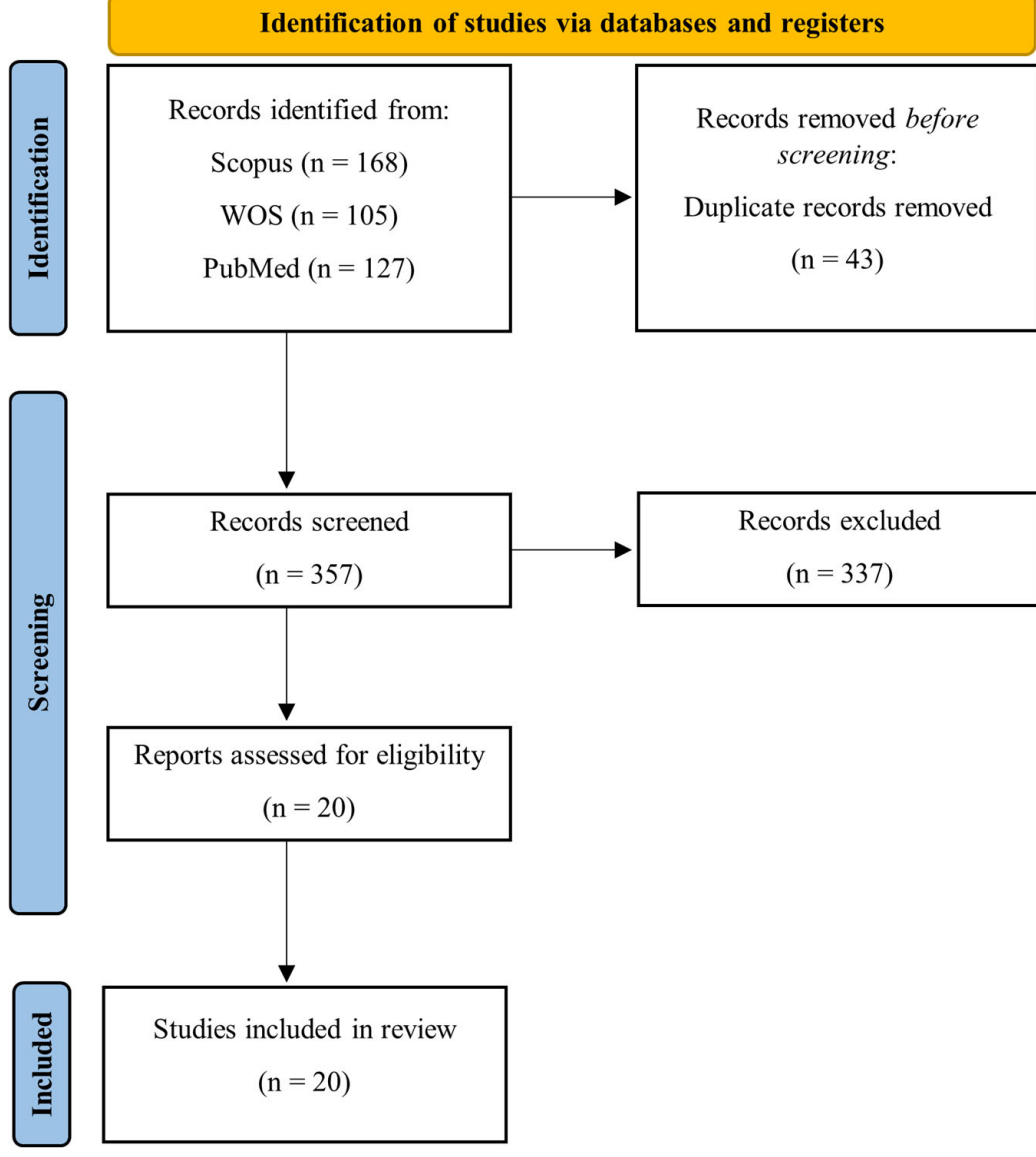
PRISMA flowchart for literature research.

The PRISMA flowchart summarizing all the screening steps of our review is reported in Figure 1. A total of 400 studies were found in the three selected databases. After removing the duplicates, the final number of papers considered eligible, satisfying all inclusion criteria, was 20.

### 3.1. Characteristics of the Studies

The main characteristics of the included studies are reported in Table 1.

#### 3.1.1. Aim of the Studies

**Figure 2:**
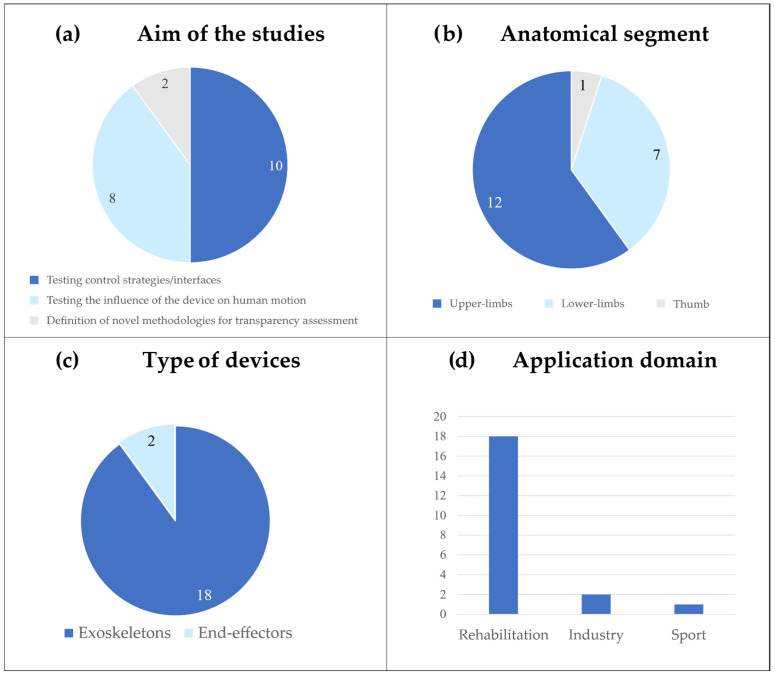
Characteristics of the studies. The graphics show the number of studies for each category. (**a**) Aim of the included studies. (**b**) Anatomical segment considered. (**c**) Type of device tested. (**d**) Application domain of the presented devices; some studies included more than one application domain.

The included studies evaluated transparency with different objectives (Figure 2), which are summarized in the following categories: (i) to assess the performance of novel control strategies [33,35,36,37,38,39,40,41] or physical interfaces [42], comparing them with existing solutions; (ii) to test the effects of different devices on human motion [43,44,45,46,47], including innovative prototypes developed by the authors [48,49,50,51]; and (iii) to define novel methodologies for transparency assessment [5,52].

#### 3.1.2. A Summary of the Devices, Application Domain, and Anatomical Segments Investigated

Twelve studies investigated upper-limb devices (Figure 2). Among them, five studies used ABLE, an active exoskeleton primarily designed for rehabilitation purposes [5,33,35,42,45]. Verdel et al. investigated both the impact of different control systems [33,35] and physical interfaces [42] on ABLE’s transparency, whereas Bastide et al. [45] evaluated the device’s transparency during the execution of simple motor tasks. In contrast, Jarrassé et al. [5] proposed a methodology for assessing human–exoskeleton interactions during the execution of upper-limb movements. Two studies tested the transparency of novel control strategies on the ARMin IV+, a robotic exoskeleton tailored for arm training in neurological patients [37,39]. Chiavenna et al. [52] examined user transparency using the passive LIGHTArm rehabilitation exoskeleton, even during arm weight support. Similarly, Pirondini et al. [43] explored the assistive modalities of the ALEx exoskeleton and their effects on transparency. Fong et al. [47] investigated how ArmeoPower, an exoskeleton designed for severely impaired individuals, influences human movements, focusing also on its applicability for evaluation purposes in clinical scenarios. Two studies presented and tested the transparency of custom-made prototypes. These included the EMU 3D Robotic Manipulandum [49], an end-effector rehabilitation device designed to assist arm movements, and a 3D-printed active elbow exoskeleton aimed at preventing musculoskeletal disorders in occupational and industrial settings [36].

Seven studies focused on lower-limb applications (Figure 2). Among these, Zanotto et al. [40] and Van Dijk et al. [41] proposed novel approaches to enhancing the transparency of ALEX II and Lopes, respectively. Both devices are treadmill-based exoskeletons aimed at improving lower-limb functions and assisting in gait training.

Similarly, Camardella et al. [38] experimentally evaluated a novel control strategy on the Wearable-Walker, a powered exoskeleton able to assist individuals with neurological impairments and enhance human capabilities in load-carrying tasks. Jin et al. [46] explored the effects of adding different weight conditions on the transparency of C-ALEX, an exoskeleton for gait rehabilitation of stroke patients. Stramel et al. [44] investigated the transparency of the mobile Tethered Pelvic Assist Device (mTPAD), an end-effector-based robotic trainer that can be coupled with a treadmill to provide pelvic assistance during gait. Two studies presented and tested prototypes developed by the authors. In their recent study, Nurse et al. [48] presented an ankle exoskeleton specifically designed for runners with the aim of preventing Achilles tendon injuries. They tested the transparency of the device during the swing phase of the gait. Conversely, Cai et al. [50] developed an active knee orthosis that aims to assist knee movements during walking.

Finally, Agarwal et al. [51] introduced and tested a novel thumb exoskeleton for the rehabilitation of neurological subjects, aiming to improve thumb mobility and enhance motor control.

#### 3.1.3. Participants

All included studies included an experimental procedure based on volunteers to assess transparency. Most of the studies recruited healthy young adults, with sample sizes ranging from 3 to 20 participants and ages typically ranging between 20 and 35 years. Only one study [50] did not involve human subjects and instead relied on a virtual simulation to test the proposed exoskeleton. The small sample sizes suggest that most of the included articles were pilot in nature, which represents a potential source of bias as it limits the generalizability of the findings and highlights the need for more robust experimental designs.

No study included clinical populations, although some devices were designed for rehabilitation purposes.

#### 3.1.4. Experimental Design

The experimental designs varied across studies, depending on the type and intended use of the device. Most protocols involved functional movements, such as reaching tasks for the upper limbs or walking sessions for the lower limbs. Transparency-related metrics were generally compared in two conditions: without the device and with the device without assistance, so in a “passive” or “transparent” modality [5,33,35,36,38,40,42,44,45,46,47,49,50,51].

**Table 1:** Characteristics of the studies included in the review.

First Author	Year	Aim	Device	Application Domain	Anatomical Segment	Subjects Included	Experimental Procedure
Nurse et al. [48]	2025	To present and experimentally evaluate an unpowered ankle exoskeleton for runners.	Custom-made prototype (Exoskeleton)	Sport	Ankle	10 healthy recreational runners (5M, 5F)	Running trials with different combinations of speeds and slopes in three configurations: without exoskeleton, with exoskeleton without assistance, and with exoskeleton with assistance.
Verdel et al. [35]	2024	To compare three different transparent controllers in movements performed outside the exoskeleton and between themselves to assess their effects on complementary performance metrics.	ABLE Exoskeleton	Rehabilitation	Upper limb	14 healthy, right-handed subjects (9M, 3F, mean age 26.33 ± 2.93 Y)	Reaching tasks in three-dimensional space. Tasks were repeated in four different conditions: without the exoskeleton and with the exoskeleton, with three different control strategies.
Souza et al. [36]	2024	To investigate whether adding motor control prediction in the controller affects transparency.	Custom-made prototype (Exoskeleton)	Industry	Upper limb	15 healthy subjects (9M, 6F, mean age 26.7 ± 2.4 Y)	Virtual pointing task in 5 different conditions: without exoskeleton and with exoskeleton, with four different control strategies.
Dalla Gasperina et al. [37]	2023	To develop a novel controller to enhance the device’s transparency. To test both quantitatively and qualitatively the proposed controller with respect to other control strategies.	ARMin IV+ Exoskeleton	Rehabilitation	Upper limb	6 healthy young participants (4M, 2F, median age 25.5 Y)	Tracking trajectories in three-dimensional space (3 planes) in a virtual-reality environment at two different speeds. The tasks were repeated for each control strategy.
Stramel et al. [44]	2022	To investigate the transparency of a lower-limb device.	Mobile Tethered Pelvic Assist Device (mTPAD) (End effector)	Rehabilitation	Lower limb	8 healthy subjects (6F, 2M, mean age 27 ± 3, 1 Y)	Three treadmill walking sessions: (i) without the device; (ii) with the device without assistance; (iii) with the device without assistance while holding the device’s frame.
Verdel et al. [42]	2022	To provide a systematic comparison of different human–exoskeleton interfaces.	ABLE Exoskeleton	Rehabilitation	Upper limb	18 healthy subjects (11F, 7M, mean age 25 ± 6 Y)	Pointing movements involving flexion/extension of the elbow in four different conditions: without the exoskeleton, with the exoskeleton, and with three different physical interfaces.
Verdel et al. [33]	2021	To improve the transparency of an upper-limb exoskeleton by developing a novel control strategy. To test the proposed control law by means of an innovative quantitative metric.	ABLE Exoskeleton	Rehabilitation	Upper limb	6 healthy, right-handed subjects (3M, 3F, mean age 25 ± 1.3 Y)	Point-to-point reaching movements in the sagittal plane, involving only elbow flexion/extension. Movements were performed without the exoskeleton and with the exoskeleton, with three different controllers.
Camardella et al. [38]	2021	To propose and experimentally evaluate a novel control algorithm for a lower-limb exoskeleton.	Wearable-Walker Exoskeleton	Industry and Rehabilitation	Lower limb	11 healthy subjects (11M, mean age 32.54 ± 5.34 Y)	Walking sessions with and without the device. When wearing the device, three different control strategies were tested. Each walking session was performed under three velocity conditions.
Chiavenna et al. [52]	2018	To evaluate user transparency through muscle synergies.	LIGHTArm Exoskeleton	Rehabilitation	Upper limb	3 healthy subjects (3M, median age 29 Y)	Functional movements for the upper limb (reaching, hand-to-mouth, and hand-to-nape) in three different conditions: (i) without the exoskeleton, (ii) with the exoskeleton without support, (iii) with the exoskeleton with weight support.
Just et al. [39]	2018	To assess the transparency of the device with two different controllers.	ARMin IV+ Exoskeleton	Rehabilitation	Upper limb	20 healthy, right-handed subjects (10M, 10F, mean age 26.2 ± 2.2 Y)	Tracking trajectories at two different speeds. The single-joint transparency study included a rectilinear path, whereas the multi-joint transparency study included a circular path. Tasks were repeated for each control strategy and at different speeds.
Bastide et al. [45]	2018	To investigate the effects of an exoskeleton on human movements in transparent modality during the execution of simple tasks.	ABLE Exoskeleton	Rehabilitation	Upper limb	18 healthy subjects (mean age 24.3 ± 5.0)	Pointing movements mainly involving elbow flexion/extension, performed with and without the exoskeletons.
Jin et al. [46]	2017	To present an improved design of the device and to investigate the effects of weight and inertia of the exoskeleton on human gait.	C-ALEX Exoskeleton	Rehabilitation	Lower limb	12 healthy subjects (9M, 3F, aged between 22 and 31 Y)	Walking sessions with different levels of added mass (0, +1.8, +3.6 kg). Each session was repeated under three conditions: (i) without the exoskeleton, (ii) with the exoskeleton without weight support for the added mass, and (iii) with the exoskeleton with weight support only for the added mass.
Fong et al. [49]	2017	To introduce the EMU robot, to test its transparency, and to discuss a controller for gravity compensation.	EMU 3D Robotic Manipulandum	Rehabilitation	Upper limb	5 healthy subjects	Reaching tasks in a virtual environment with and without the robot.
Cai et al. [50]	2017	To propose and virtually evaluate a novel design of a lower limb exoskeleton to enhance transparency.	Custom-made prototype (Exoskeleton)	Rehabilitation	Lower limb	Virtual human model	Walking sessions with and without the exoskeleton.
Agarwal et al. [51]	2017	To design a novel thumb exoskeleton and to experimentally evaluate its workspace and kinematic transparency.	Custom-made prototype (Exoskeleton)	Rehabilitation	Thumb	4 healthy subjects (3M, 1F, aged between 20 and 33 Y)	Four different thumb movements exploring full range of motion at four different speeds. Movements were performed with and without the exoskeleton.
Pirondini et al. [43]	2016	To evaluate the possible use of the exoskeleton for rehabilitation and to investigate the effects of different assistive modalities.	ALEx Exoskeleton	Rehabilitation	Upper limb	6 right-handed healthy young subjects (5M, 1F, mean age 26.5 ± 3.4)	Three sessions performed: (i) reaching movements without and with the exoskeleton without assistance; (ii) reaching movements in transparent and assistive modality; (iii) reaching movements in transparent and assistive modality with two different control strategies.
Fong et al. [47]	2015	To evaluate the effect of an exoskeleton on human movement and to check the accuracy of the data provided by the robot with respect to external sensors.	ArmeoPower Exoskeleton	Rehabilitation	Upper limb	10 healthy subjects (mean age 28.2 ± 6.1)	Reaching tasks in a three-dimensional virtual environment with and without the robot.
Van Dijk et al. [41]	2013	To propose and experimentally evaluate novel controllers to improve the transparency of a device.	Lopes Exoskeleton	Rehabilitation	Lower limb	4 healthy subjects (4 males, mean age 28 ± 2)	Walking sessions with the exoskeleton with different control strategies. The walking sessions were repeated at two different speeds.
Zanotto et al. [40]	2013	To propose and experimentally evaluate a novel approach to optimize the transparency of a device.	ALEX II Exoskeleton	Rehabilitation	Lower limb	3 healthy subjects (3M, mean age 28 ± 1 Y)	Four walking sessions: (i) and (iv) without the robot; (ii) and (iii) with the robot, with two different controllers. Sessions (i) and (iv) were split into two sub-sessions, one completely free and the other with the robot’s orthoses attached to the leg.
Jarrassé et al. [5]	2010	To propose a methodology to evaluate human–robot interactions.	ABLE Exoskeleton	Rehabilitation	Upper limb	10 healthy subjects (9M, 1F, aged between 22 and 30 Y)	Pointing movements in 3D space were performed with and without the exoskeleton.

Three studies extended their approach by also comparing different assistive modalities, assessing transparency-related indicators without the device and with the device under varying levels of assistance [43,48,52]. In contrast, three studies focused exclusively on comparing different control strategies implemented for the tested device, without including a baseline recording without the device [37,39,41]. Further details on the experimental procedures are listed in Table 1.

### 3.2. Transparency Definition and Assessment

Transparency definitions and assessment metrics are summarized in Table 2.

#### 3.2.1. Transparency Definitions

**Figure 3:**
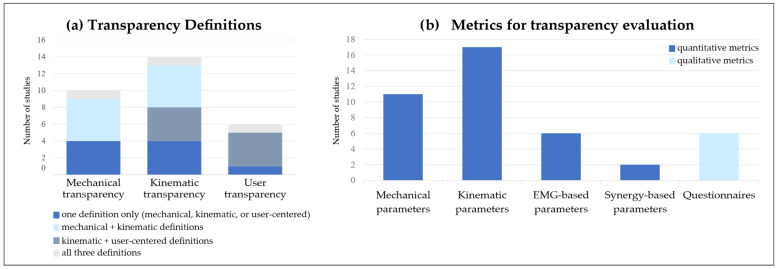
Characteristics of the studies. (**a**) Transparency definitions adopted. Blue bars represent studies that used only one definition, either mechanical, kinematic, or user-centered. Light-blue bars indicate studies that combined mechanical and kinematic definitions. Dark-gray bars refer to studies including both kinematic and user-centered definitions. Light-gray bars correspond to studies that incorporated all three definitions. (**b**) Metrics included for transparency evaluation. Blue bars indicate quantitative metrics, while light-blue bars represent qualitative metrics.

The definitions of transparency adopted in the selected studies can be grouped into three main categories (Figure 3): (i) *mechanical transparency*, which is usually considered an intrinsic characteristic of the device being tested, such as the absence of undesired forces or resistance when the device is not actively assisting; (ii) *kinematic transparency*, which is defined based on the absence of disturbances in movement execution. In this case, transparency is assessed by comparing human motion patterns with and without the device to determine whether it affects natural kinematics; and (iii) *user transparency*, which relates to the user’s perception and motor control. This definition emphasizes the subjective experience of the user and how much the device influences motor strategies, perceived effort, or sensory feedback during task execution.

Across the selected studies, five relied solely on the mechanical definition [37,39,40,41,49], while four focused exclusively on kinematic transparency [44,45,46,51]. Five studies included both mechanical and kinematic definitions to provide a more comprehensive evaluation [5,33,35,36,49]. User transparency was considered in six studies: one study adopted it as the main definition [52], whereas four studies included it in combination with kinematic transparency [38,43,48,50]. Only one study included all three definitions to provide a more comprehensive and complete evaluation of transparency [42].

#### 3.2.2. Metrics for Transparency Assessment

**Figure 4:**
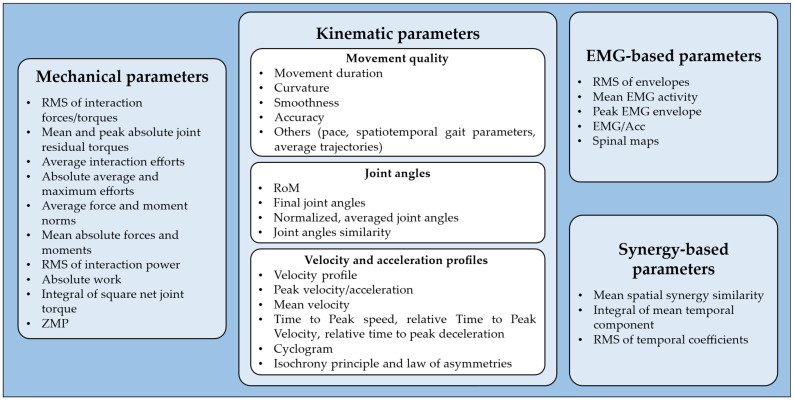
Summary of the parameters used for transparency assessment.

The metrics used in the selected studies to evaluate transparency can be classified into several categories, which will be presented in the following sections and summarized in Figure 3 and Figure 4.

##### Mechanical Transparency

Eleven studies assessed transparency by using the robot’s mechanical parameters, primarily obtained through force/torque (F/T) sensors or inverse dynamics. Most of these studies focused on evaluating interaction forces and torques at the human–device interface. A commonly used metric was the root mean square (RMS) of the interaction effort over a single trial, which was employed by four studies [36,38,40,41]. Among these, Souza et al. and Zanotto et al. [36,40] also reported the maximum absolute value within each trial, offering insights into peak mechanical disturbances. Two studies [37,39] evaluated the mean and peak absolute residual torques at the joints, providing useful information on the mechanical load imposed by the device on the joints during interaction and movement execution.

In a recent work, Verdel et al. [35] reported the average interaction efforts across participants, using them as qualitative, global indicators of interaction and transparency. In addition, they also included the absolute average and the absolute maximum interaction efforts to assess long-term and short-term device acceptability, respectively. Jarrassé et al. [5] measured the average force and moment norms at each interaction point, along with the mean absolute values of the individual force and moment components recorded by each F/T sensor used in the experiment. Van Dijk et al. [41] estimated the RMS of the interaction power, which reflects the energy exchange between the robot and the user, as an indicator of whether the robot was supporting or resisting movement.

A different approach was adopted by Bastide et al. [45], who used inverse dynamics to compute the absolute work at the elbow joint, measured as the integral of the absolute joint power and the integral of the squared net joint torque. Finally, in their simulation-based study, Cai et al. [50] assessed joint torques and the evolution of the zero moment point (ZMP), a critical parameter for evaluating balance and stability.

##### Kinematic Transparency

Seventeen of the included studies considered a wide range of kinematic parameters (Figure 3). For more clarity, the parameters are hereby grouped into sub-categories based on the aspect of movement they describe.

- *Metrics based on movement quality*. Five studies assessed movement duration, defined as the time difference between movement onset and offset [5,33,35,36,45]. Four studies analyzed curvature, measured as the maximum deviation from a straight trajectory between the starting and ending points [5,35,47,49]. A similar metric, referred to as mean distance, was used by Pirondini et al. Mean distance was computed as the mean absolute distance between the trajectory and the straight line connecting the start and end points, normalized to the length of the ideal path [43]. Six studies evaluated movement smoothness using different approaches: spectral arc length (SPARC) [36,37,47,49], jerk [5], cross-correlation between trajectories in each trial [38], and the number of peaks of the velocity profile [43]. Movement accuracy was assessed in four studies. Fong et al. [47,49] defined accuracy as the shortest distance between the cursor and the virtual target. Dalla Gasperina et al. [37] implicitly assessed movement accuracy by means of the root mean squared error (RMSE) between ideal and actual trajectories, while Souza et al. [36] considered accuracy as the maximum size of the overshoot, defined as the largest deviation beyond the ideal target during movement execution before correction and stabilization. Other indicators of movement quality for transparency assessment included pace, defined as the difference between the actual speed and the speed required to follow the metronome [43], average movement trajectories across participants [35], and, in lower-limb application, spatiotemporal gait parameters such as step length, stride time, and step width [38,40,44,46].
- *Metrics based on joint angles*. Five studies included joint angular excursions, i.e., the range of motion (RoM), to compare different conditions [5,40,43,46,48]. Jarrassé et al. [5] considered only the final joint angles, calculated at the time instant when the pointer touches the selected target, whereas Zanotto et al. [40] computed the normalized, averaged joint angles across conditions. Other works evaluated joint angle similarity more globally: Agarwal et al. [51] applied correlation analysis for a quantitative approach, whereas Cai et al. [50] conducted only a qualitative assessment.
- *Metrics based on velocity and acceleration*. Two studies provided a qualitative analysis of velocity profiles, aiming to identify deviations from the typical bell-shaped profile observed in unconstrained reaching movements [5,42]. Other studies focused instead on quantitative parameters such as peak velocity [33,35,42,47,49], peak acceleration [35,42], and mean velocity [45]. In both studies by Fong et al. [47,49], time to peak speed was analyzed, which might be useful for identifying shifts in the temporal structure of the movement. Bastide et al. [45] introduced the relative time to peak velocity (TPV), defined as the ratio between acceleration duration and total movement duration. Similarly, Verdel et al. [42] computed the relative time to peak deceleration, defined as the ratio between the time to peak deceleration and the overall movement duration. This metric is particularly useful for capturing local alterations towards the end of human movement. Additional metrics are the cyclogram of shoulder angular velocity plotted against elbow angular velocity, presented by Jarrassé et al. [5], which highlights joint synchronizations and reveals alterations in inter-joint coordination. Beyond these metrics, some studies have explored high-level motor control principles. For instance, the isochrony principle suggests that individuals adjust movement speed as a function of the distance to be traveled, to maintain a consistent movement duration [45,53]. Specifically, this principle was tested by evaluating the linear relationship between movement amplitude and velocity and assessing whether it is preserved when interacting with the device [33,45]. Finally, Bastide et al. [45] tested the law of asymmetries, which states that acceleration time is typically longer in downward movements compared to upward ones of a similar duration. In this context, asymmetry was quantified as the difference in TPV between upward and downward movements.

##### User Transparency

User transparency is mostly based on electromyography (EMG)-based parameters. It has been investigated less extensively than kinematic transparency. In fact, only six studies included quantitative indicators based on recorded EMG signals (Figure 3).

Three studies computed the RMS of normalized EMG envelopes, which reflects the average muscle effort exerted by participants [35,42,43]. Similarly, Nurse et al. [48] calculated the mean EMG activity of the tibialis anterior during the swing phase of gait, as the activation phase of this muscle was of specific interest to test the transparency of an ankle exoskeleton prototype. Stramel et al. [44] extracted the peak EMG value for each experimental condition, aiming to quantify the maximum activation level reached by each muscle. In a more analytical approach, Verdel et al. [33] proposed a novel index named “EMG/Acc”, designed to capture the relationship between motor command and the resulting acceleration. This parameter is defined as the ratio between the RMS activation of the agonist muscle and the maximal acceleration achieved during movement, normalized to the same value obtained in the baseline condition without the device. The index is computed as follows [42]:

(1) $$\frac{EMG}{Acc}=\frac{{PA}_{B}}{{RMS}_{ag,B}}\left(\frac{{RMS}_{ag}}{PA}\right)-1$$

where *RMS_ag_* is the mean *RMS* activation of the agonist muscle, *PA* is the peak acceleration of the movement, and subscript *B* denotes the corresponding values recorded in the no-device baseline condition. This metric could be seen as an indication of the level of efficiency: by linking muscle activation to the resulting acceleration, the *EMG/Acc* index provides insights into how the device may interfere with the user’s motor planning and execution [33,42]. An in-depth approach was proposed by Pirondini et al. [43], who reconstructed spinal maps to estimate motoneuronal activity in the spinal cord. This method provides a spatiotemporal representation of spinal activation during movement execution, offering the possibility to assess whether different assistance modalities, or simply the interactions with the device, affect spinal cord activity.

##### Synergy-Based Parameters

Only two studies included muscle synergy analysis as a method for transparency assessment (Figure 3). Pirondini et al. [43] extracted muscle synergies to investigate whether the use of the exoskeleton influenced muscle coordination during different assistance modalities. Specifically, they identified synergies separately for each condition and assessed their similarity by computing the scalar product between corresponding spatial weights. In addition, they computed the RMS of the temporal coefficients to compare the level of activity of each muscle synergy across conditions. Similarly, Chiavenna et al. [52] evaluated the mean spatial synergy similarity to detect any potential alterations in muscle coordination patterns. To investigate the effects of weight support, they also computed the integral of each mean temporal component (MTC), which served as an indicator of the activation magnitude associated with each spatial synergy.

##### Questionnaires

Six studies incorporated questionnaires to gather user feedback, providing insights into their experience with the device. In four studies, questionnaires were used to evaluate the perceived level of comfort and sense of freedom while using the device, reflecting the user’s perception of transparency [35,39,42,48]. In contrast, Souza et al. [36] used questionnaires to ask participants to rank different controller options. Although this approach allows for capturing user preferences, it does not directly assess the perceived transparency of the device. Finally, Dalla Gasperina et al. [37] incorporated both types of questions, providing a more comprehensive assessment of user perception.

### 3.3. Sensitivity Analysis

Based on our qualitative sensitivity analysis, we identified the five most relevant studies, as those that had higher reliability, evaluated through a set of indicators, including the number of citations per year, the number of participants involved, and the statistical robustness of the results, i.e., whether the findings were supported by a structured statistical analysis (Table 3). Despite focusing on a smaller subset of works, the conclusions remained consistent with those previously discussed for the whole group of studies. In fact, all selected articles in this subgroup adopted different definitions of transparency and employed various quantitative metrics for its assessment. Interestingly, both Pirondini et al. [43] and Verdel et al. [42] included the concept of user transparency. Moreover, Pirondini et al. [43], which is the most cited study, stands out since it incorporates muscle synergy analysis. The fact that the most influential work is also one of the few to explore this aspect highlights the growing interest in using motor control measures to evaluate transparency. This further supports the incorporation of non-mechanical variables into transparency assessment frameworks.

## 4. Discussion

This systematic review examines the concept of transparency in human–device interaction and how it is evaluated in current research. Although the reviewed studies offer valuable insights, several key aspects remain unclear or underexplored. In the following section, we present a critical analysis of the main findings of our screening, highlighting the relevance of transparency evaluation, key limitations in the existing literature, and directions for future research.

### 4.1. Main Fields of Application

Most of the reviewed studies focused on the evaluation of transparency in exoskeleton devices. Since exoskeletons are becoming progressively more common in both rehabilitation and industrial settings, they are often prioritized in the literature. However, transparency is equally important for any device that physically interacts with users. Transparency is a broad concept that is not limited to exoskeletons alone, but also applicable to a variety of assistive technologies, including orthoses, end-effectors, and collaborative robots. This emphasis on exoskeletons is likely due to the fact that they completely enclose the body segment, making transparency not only desirable but also essential for their effective use. Most of the studies (13 out of 20) evaluated transparency in prototypes, indicating that this metric is primarily used as an exploratory tool to characterize a device during the design phase. This is fundamental, as it provides insights that can guide improvements in device design and development before commercial production. Nonetheless, evaluating commercially available devices would be valuable for understanding how they affect user movement in real-world scenarios. In terms of application domains, most of the studies focused on rehabilitation, reflecting the strong clinical interest in minimizing undesired effects that may compromise motor recovery in neurological subjects. In this context, transparency is closely linked to the effectiveness of rehabilitation interventions. Similarly, in industrial applications, transparency significantly influences device usability and comfort, both in short-term and long-term use. Despite its relevance, transparency has been only marginally investigated in non-clinical domains such as daily-life assistance, industrial support, and sport performance enhancement. As only a few works have investigated these areas of research, they mainly remain underexplored, highlighting the need for further investigations. Industrial exoskeletons are typically designed to assist highly repetitive tasks, in non-neutral postures, and with heavy workloads [54], with the primary goal of reducing muscular effort and fatigue. Their effectiveness is commonly evaluated through reductions in joint torque or muscular activity [55]. From the user perspective, assessments often focus on ergonomics to prevent work-related MSDs (WRMSDs) [56]. These devices are prevalent in the automotive industry, where they are used to support the upper limbs [57]. A recent review of industrial exoskeletons highlighted that EMG and kinematic analyses are commonly used for assessing device efficacy (i.e., strain reduction), while scales and subjective questionnaires are used to evaluate perceived discomfort and ergonomics [58]. However, even biomechanically efficient exoskeletons may lack usability and comfort, underscoring the importance of evaluating transparency in industrial contexts. Indeed, the existing literature suggests that while exoskeletons can effectively reduce physical strain, there is still a need for improved designs to enhance comfort, usability, and biomechanical compatibility for long-term use [59,60].

### 4.2. A Set of “Transparency” Definitions and an Ambiguous Concept of Transparency

The literature reveals a notable lack of consensus on the definition of “transparency”. While most studies agree on a general concept of transparency—typically described as the absence of undesired interference from the device [6,31]—each study interprets this high-level concept differently. In most cases, transparency is considered an intrinsic, mechanical property of the device. From this perspective, a device is considered transparent if it minimally resists or disturbs the user’s intended motion, especially when not actively providing assistance. However, this definition just considers the mechanical aspects of the complexity of human–robot interaction, since it fails to account for user perception and for how the device affects motor control. Moreover, the terminology itself is not consistent in the literature: in some cases, the term “transparency” is not explicitly mentioned, but instead, mechanical measures such as “backdrivability” [8,61,62] and “compliance” [25,63,64] are used interchangeably as synonyms. Although these characteristics can be useful for a general understanding of a device’s mechanical behavior, they are insufficient to completely define transparency, especially in human-centered applications. Mechanical features such as high backdrivability, low inertia, and compliant actuators may contribute to higher transparency, but they do not guarantee that the user perspective and experience of the interaction are perceived as transparent. In fact, a system that performs well from a mechanical standpoint might still be perceived as non-transparent. For this reason, terms such as backdrivability, compliance, and low impedance were not examined in this review. Although they are often used as synonyms to denote transparency, these terms tend to narrow the focus to purely mechanical aspects. In contrast, the aim of this study is to explore a broader notion of transparency; rather than focusing solely on mechanical properties, this study adopts a more comprehensive perspective that also includes kinematics aspects and the perceptual experience of the users. On the contrary, several studies considered in part the user perspective, without referring to the concept of transparency.

Across the selected studies, only a few explicitly included a more comprehensive definition of transparency, focusing on the transparency perceived by the subjects. For instance, Chiavenna et al. [52] defined transparency by means of muscle synergies, which might reflect alterations in motor control strategies induced by interaction with the device. According to this view, a transparent device should preserve natural movement patterns and avoid interfering with the user’s sensorimotor control. This implies that users should not experience any constraints or disturbances or be forced to modify their motor control strategies to compensate for the presence of the device. This feature is especially critical in robots designed for neurorehabilitation. In synergistic control, this concept may correspond to unaltered spatial synergies when using the device, as suggested by Chiavenna et al. [52]. In a similar way, Pirondini et al. [43] suggested that transparency is achieved when movement kinematics and neuromuscular strategies (i.e., muscle activation patterns and their coordination) are both preserved during device use.

Therefore, a comprehensive definition of transparency should integrate not only high-level kinematic and mechanical factors such as the absence of undesired interaction forces or alterations in the user’s natural movement, but also more in-depth aspects, including the user’s subjective feelings and the preservation of natural motor control strategies. In our view, transparency is a multifaceted concept that cannot be fully captured by a single-domain definition. Consequently, future studies should avoid focusing exclusively on one perspective over others, but rather adopt a multidimensional approach that combines mechanical, kinematic, and user-centered aspects.

### 4.3. Current Strategies for Enhancing Mechanical Transparency

In the literature, transparency is often considered primarily as a mechanical, programmable property of the device, which can be optimized through a combination of software-based control strategies and hardware innovations. From a control perspective, several techniques such as impedance and admittance control [65,66] are widely adopted to minimize the device’s resistance and enhance its ability to adapt to the user’s movements. More specifically, impedance control regulates the dynamic relationship between position and force by generating a compliant torque or force output in response to user-applied displacements. Conversely, admittance control takes external forces as inputs to generate appropriate motions, usually in terms of position or velocity [66]. Admittance control is particularly suitable when dealing with devices with stiff or non-backdrivable actuators. Despite their differences, both strategies aim to reduce the perceived stiffness of the device, thereby enhancing mechanical transparency during human–robot interaction. In addition to these well-established methods, recent advances have explored novel control approaches that promote more user-centered interactions, following the principle of Human-in-the-Loop optimization [30]. For instance, EMG-driven controllers aim to predict the user’s motor intention by acquiring muscle activity in real time, improving the device’s responsiveness to the user and allowing for more transparent interactions [67,68]. Similarly, adaptive control strategies aim to improve transparency by continuously adjusting the device’s properties based on the user’s current state, which is usually achieved by integrating biosensors in the control loop [68].

On the hardware side, solutions such as series elastic actuators (SEAs) or model-based compensation have proven to contribute to enhancing mechanical compliance and the responsiveness of the device [66,69,70]. Together, these approaches aim to reduce mechanical impedance and promote what is commonly referred to as “transparent” behavior. Moreover, a promising approach for future research regards soft wearable robots and exosuits, which have emerged as alternative solutions to traditional rigid devices, as they are designed to interact with the body in a more compliant and natural manner. These devices rely on flexible structures and soft materials to deliver assistance while minimally constraining the user’s natural movements [8,71]. Indeed, soft wearable robots may offer higher transparency compared to rigid exoskeletons, thanks to their lightness, higher adaptability, and flexibility. Their compliant structures typically interfere less with the user’s natural movements, resulting in improved comfort and reduced perceived effort. Additionally, the use of soft materials may minimize interference with external measurement systems, thereby enhancing both the accuracy and comfort of assessment sessions. However, from a control perspective, soft robots present significant challenges: the non-linear behavior of soft materials, along with the complexity of human–robot interaction forces, makes it more difficult to accurately model and fine-tune the system’s response.

However, despite improving the mechanical performance of the devices, these strategies should be complemented by approaches that also account for the user’s point of view. Mechanical transparency, though useful, is not sufficient on its own to ensure a natural human–device interaction. Moreover, assessing transparency solely through interaction forces and joint torques fails to capture user perception or the neuromuscular adjustments that may arise. Therefore, a comprehensive understanding of transparency should go beyond purely mechanical aspects, incorporating user experience into the evaluation process.

### 4.4. The Trade-Off Between Transparency and Assistance and Its Role in Motor Learning and Neurorehabilitation

In the context of motor learning and rehabilitation, transparency is a key factor in encouraging active patient participation during movement execution. According to the schema theory [72], motor relearning is more effective when individuals experience and learn from the effort–error relationship [73]. Therefore, patients must be actively involved in both motor planning and movement execution, and the device must avoid interfering with these processes. In this regard, transparency plays a fundamental role: a non-transparent device might alter physiological motor control, preventing users from experiencing their own motor errors and potentially leading to maladaptive neuroplasticity. In such cases, physiological motor functions may be replaced with compensatory, abnormal movements, ultimately reducing therapy effectiveness [74].

Building upon this concept, an important challenge is to balance assistance and transparency throughout the rehabilitation process. While high levels of assistance are often necessary in the early stages, particularly for patients with severe motor impairments, assistance should be progressively reduced as patients regain motor functions [6]. Excessive assistance might hide movement errors that patients experience, reducing their volitional effort and ultimately limiting the effectiveness of the rehabilitation treatment. It has been suggested that long-term functional improvements, including the so-called carry-over effect, are more likely to occur when patients are actively engaged in movement planning and perceive the device as integrated in their control loop [75]. To address this, many rehabilitation strategies incorporate assistance-as-needed approaches, in which the device only intervenes when the user is unable to complete the task autonomously [68]. A common example is tunnel-based control, in which the system defines an ideal trajectory (i.e., the “tunnel”) and allows the patient to perform the movement independently. The device only intervenes if the user significantly deviates from the trajectory or takes too long to complete the task [6,66]. These strategies aim to support task performance without reducing active participation, maintaining a good level of transparency, and ensuring successful task completion.

Even within the selected literature, transparency clearly emerges as a desirable feature in READs to promote safe and effective interaction with the user. Ideally, when not actively providing assistance, the device should interfere as little as possible with the user’s natural movement and motor control. However, during active assistance or support, some degree of interaction is inevitable and even necessary to effectively empower the user; thus, the device naturally becomes non-transparent. In this context, a key factor for promoting motor recovery lies in understanding which mechanisms of motor learning are elicited in such phases. Despite its significance, this trade-off between transparency and empowerment is almost uninvestigated in the literature. This gap limits the possibility of fully capturing human–device interaction and hinders the development of more tailored rehabilitation strategies for individuals with neurological impairments. Indeed, most of the investigated studies evaluated transparency exclusively under zero-assistance conditions, overlooking scenarios in which assistance or support are actively provided. Only three studies explicitly included this aspect. For instance, Nurse et al. [48] tested an ankle exoskeleton during both non-assistance and assistance modes, where the device assisted the Achilles tendon during the loading phase of running. Chiavenna et al. [52] investigated the effects of weight support on upper-limb functional movements using a non-actuated exoskeleton, while Pirondini et al. [43] compared muscle activity and coordination of reaching movements across three conditions: without the exoskeleton, with the exoskeleton without assistance, and with the exoskeleton providing assistance. These studies highlight the importance of evaluating transparency in assisted conditions to better understand the dynamics of user–device interaction. However, they remain isolated cases, and their findings are not sufficient to draw general conclusions. On the other hand, several studies in the literature have investigated how assistance and support influence muscle synergies and movement performance, primarily to assess the benefits of empowerment [76,77,78,79]. Although these studies offer valuable insights into how assistance or support influences motor control, they do not explicitly refer to the concept of transparency. This omission is probably due to the lack of a clear and globally accepted definition of the term, which makes it challenging to interpret these findings within the context of transparency, thus introducing potential bias. This ultimately limits the possibility of building a complete understanding of human–device interaction, thereby preventing the development of standardized methodologies for transparency assessment. Moreover, even though the need to balance transparency and assistance is widely acknowledged, specific metrics to quantitatively assess this trade-off are still lacking. Most studies evaluate transparency and empowerment separately, without integrating them into a unified framework. This represents a significant gap in the current literature, as the development of such metrics could deepen the understanding of human–robot interaction and support the design of more effective READs and control strategies.

### 4.5. A Variety of Metrics for Transparency Assessment

Despite significant advancements in the development of READs and their growing adoption across various application scenarios, there are currently no standardized guidelines for quantitatively evaluating transparency and user perception. This lack of consistency hinders direct comparison between different devices and limits the generalization of findings across studies. One of the main reasons behind this issue is the absence of a globally accepted definition of transparency: without a clear and shared conceptualization, it becomes difficult to establish standard parameters for measuring it. As a result, the literature reveals considerable variability in the adopted evaluation metrics, with no clear consensus on which measures should be considered essential.

Our screening revealed that transparency is primarily assessed through high-level parameters, such as interaction efforts, movement quality indicators (e.g., smoothness, curvature, duration, and accuracy), RoM, and peak velocity. These metrics strongly align with the most common interpretation of transparency as the absence of movement alterations and undesired interaction forces. However, these metrics exclusively focus on objective, mechanical, or kinematic aspects, largely neglecting the subjective perception of the users. Therefore, it is crucial to integrate these indicators and propose more comprehensive evaluation protocols that are able to systematically capture user experience and perception.

Another critical aspect concerns the instrumentation used for data collection. Many of the currently adopted metrics depend on measurements obtained through external sensors, such as inertial measurement units (IMUs) or surface EMG systems. However, the use of bulky sensors can introduce practical limitations, particularly in industrial applications or while using certain types of exoskeletons. Bulky sensors may physically interfere with the device’s structure, which might introduce noise and artifacts into the collected data, thus reducing the accuracy and reliability of the measurements. Moreover, the wearability of these sensors may lead to user discomfort during device donning and after prolonged use, negatively impacting usability and user acceptance. None of the selected studies directly addressed this problem or proposed a solution for mitigating this issue; however, bulky sensors may reduce the transparency of the device. These challenges must be considered when defining standard evaluation protocols, as they may limit the applicability of certain measures.

### 4.6. User Transparency: Limitations of Conventional Metrics and the Role of Synergistic Control

As already discussed, transparency in READs is often assessed using mechanical metrics such as interaction forces and torques. Kinematic parameters such as movement smoothness, duration, and accuracy are also frequently used, since they are simple to collect and interpret. However, they are high-level indicators that do not provide detailed insights into the underlying motor control strategies. In fact, a movement that appears smooth or nearly unaltered when using a device might suggest perfect transparency. However, this might instead result from altered motor commands from the central nervous system (CNS) to compensate for the device’s influence. In such cases, the user may be unconsciously modifying muscle activation patterns to maintain similar kinematic outcomes. Therefore, apparent transparency at the kinematic level might hide significant changes in motor control.

Subjective questionnaires are often the only way to assess transparency from the user’s perspective. However, no standardized questionnaires currently exist to measure users’ perception of transparency. Instead, several works have adopted heterogeneous sets of questions and rating scales, often tailored to the specific experimental context of the study, to evaluate aspects such as perceived effort, comfort, accuracy, and control. In the literature, several standardized questionnaires are commonly employed to evaluate user experience in the context of human–device interaction. These include the Borg Rating of Perceived Exertion (RPE) [80], which assesses the perceived physical effort; the System Usability Scale (SUS) [81], which evaluates the perceived usability of the technology; and the Technology Acceptance Model (TAM) [82], which explores aspects such as the perceived ease of use. While these instruments provide valuable information on the global level of user experience, none of them is specifically designed to assess transparency. Nevertheless, they could serve as a solid foundation for the development of a more comprehensive and targeted tool aimed at evaluating users’ perceptions of transparency. Despite their relevance, these subjective assessments do not offer objective evaluations or physiological information on how users adapt to the device.

Therefore, using evaluation tools that can directly assess motor control strategies is essential to achieving a more comprehensive and human-centered assessment of transparency. A promising approach in this direction involves the analysis of synergistic control, specifically muscle synergies, which represent the coordinated activation of groups of muscles that work together to execute specific movements or perform particular tasks. According to the muscle synergy theory, the CNS simplifies motor control by recruiting and coordinating multiple muscles as functional units or modules [83,84,85]. Over the last few decades, several synergy models and computational methods have been proposed to describe how muscle activations are coordinated, namely spatial [86], temporal [87,88], or spatiotemporal [89] models. For instance, in the spatial synergy model, also referred to as the time-invariant or synchronous model, muscle synergies are defined by fixed sets of muscle weights, which remain constant across time and conditions. These synergies are modulated by task-dependent, time-varying coefficients, which reflect how each synergy is recruited during movement execution [90].

By examining how these motor modules change when using READs, synergy analysis can provide a more in-depth understanding of human–device interactions. For instance, alterations in synergy structure may suggest that users are adapting their motor control strategy to compensate for the interaction with the device. This was investigated in the studies by Chiavenna et al. [52] and Pirondini et al. [43], which explored how upper-limb muscle synergies are affected by the interaction with an exoskeleton providing weight support and active assistance, respectively. Their findings suggest that a transparent device should preserve the user’s original motor control strategy: ideally, in zero-assistance mode, no changes should occur in the synergy structure, and only minimal variations in temporal coefficients should be observed. An increase in temporal activations might occur when interacting with a non-transparent device or when using a device during resistive modality [6,66], reflecting additional muscular effort.

This highlights the importance of assessing transparency not only in zero-assistance conditions but also during assisted or supported movements. In this context, the trade-off between transparency and empowerment becomes evident: while assistance is intended to reduce muscular effort, it should not compromise the natural structure or timing of motor control. A transparent device is therefore expected to preserve the spatial synergy structure and show only a reduction in temporal activation profiles, which aligns with the reduced muscular effort. In contrast, changes in synergy structure suggest motor control adaptations, pointing to a loss of transparency. Moreover, the integration of kinematic–muscle synergy analysis [91] could provide a physiological interpretation of muscle synergies and offer additional insights into transparency assessment. Despite its potential, synergy analysis also presents some limitations that should be acknowledged. First, reliable synergy extraction typically requires a high number of EMG sensors, which increases system complexity, as well as multiple repetitions of stereotyped movements, which may not be feasible in all experimental conditions. Additionally, the mapping between muscle synergies and the corresponding motor output is not always straightforward, thus limiting results interpretability. However, recent approaches such as the Mixed Matrix Factorization (MMF) algorithm [91] may help address this issue by integrating muscle and kinematic information for a more physiological interpretation of synergies.

Overall, these findings highlight the potential of synergy analysis for assessing the level of transparency of READs, supporting a more detailed evaluation of human–device interaction. This approach is not only relevant for optimizing rehabilitation outcomes, but also for promoting safe use of READs in industrial settings, where preserving natural muscle coordination can help prevent WRMSDs, enhancing comfort and long-term usability.

To move towards a more reliable benchmarking framework, it is necessary to establish a set of core metrics that are essential for transparency assessment. These should include both objective parameters, such as movement quality indicators or interaction efforts, and subjective measures related to user perception and motor control. A comprehensive assessment of transparency should address all three domains: the mechanical domain, through the analysis of interaction forces and torques; the kinematic domain, to assess movement alterations; and the user perception domain, through subjective questionnaires and motor control analysis (Table 4). In this regard, synergy-based metrics might offer valuable insights into motor adaptation and control, while standardized questionnaires could support a more structured evaluation of user experience. In addition, a set of accessory metrics could be defined, allowing the capture of additional information that might be relevant for specific devices, tasks, or applications. We acknowledge that including all three domains might not always be feasible in all experimental conditions. However, this framework should serve as a reference for future studies aiming to evaluate transparency in a comprehensive way. When full-domain assessment is not feasible, studies should clearly state which domain(s) were considered.

Organizing these core and accessory metrics into a shared and structured taxonomy would represent a first step towards building a comprehensive evaluation approach. This would not only enhance the comparability of results across studies but also provide a more complete understanding of human–device interaction.

### 4.7. Limitations and Future Works

This review has some limitations that should be acknowledged. First, the heterogeneity of the included studies, particularly in terms of evaluation methods, limited the possibility of drawing general conclusions. Moreover, the absence of a standardized definition of transparency and the inconsistent use of terminology further complicated direct comparison across studies. Despite these challenges, the review offers a structured overview of current trends and gaps in transparency evaluation in robotic and assistive devices. Another limitation is the participant population. All selected studies investigated transparency exclusively in healthy young participants, limiting the representativeness of their findings. Given the broad relevance of transparency in rehabilitation, the lack of data on elderly people and patients is surprising; however, the focus on healthy young participants may have been intended to define a baseline before applying the same measures to patients. Future research should involve a more diverse population to provide a more comprehensive assessment of transparency. Additionally, studies have mainly focused on clinical and rehabilitative applications, with only limited exploration of the industrial field. However, transparency is equally important in industrial settings not only to reduce physical strain and prevent WRMSDs, but also to ensure comfort and usability during prolonged use.

Future research should focus on defining and adopting a shared standardized framework for transparency definition and assessment in human–device interaction, allowing for more consistent and comparable evaluations. Additionally, transparency should be assessed not only in zero-assistance conditions, but also during active assistance or support, where the trade-off between transparency and empowerment becomes critical. Finally, the integration of more in-depth measurements, such as muscle synergy analysis, could provide a deeper understanding of how READs affect motor control and user experience.

## 5. Conclusions

This review highlighted the central role of transparency in the evaluation of READs. Although transparency is widely recognized as a desirable feature, its definition and assessment remain inconsistent across studies. Current evaluations predominantly focus on mechanical and kinematic parameters, often neglecting the user’s perspective and the underlying motor control adaptations. To move toward more comprehensive and user-centered approaches, future research should integrate user-centered measures, such as synergistic control, to better reflect the user’s experience and physiological response. Moreover, establishing a shared definition and standardized metrics for transparency assessment is essential for achieving a deeper understanding of human–robot interaction and enabling comparisons across studies and devices.

## Figures and Tables

**Table 2:** Transparency evaluation in the included studies.

First Author	Year	Transparency Definition	Anatomical Segment	Quantitative Metrics	Questionnaires	Main Results
Nurse et al. [48]	2025	Capability of the device to not interfere with human movement or cause discomfort or fatigue in the user.	Ankle	Kinematic parameters: ankle RoM. EMG parameters: average tibialis anterior muscle activity during the swing phase (mean EMG value during the average swing phase).	Participants were asked to rate a statement concerning the exoskeleton’s lack of restriction of ankle motion during the swing phase.	No significant effects were found on RoM or muscle activity. In total, 6 out of 7 participants reported that they did not feel impeded by the exoskeleton during the swing phase.
Verdel et al. [35]	2024	Capability of the device to follow human movement without altering it, which ultimately results in null interaction efforts.	Upper limb	Mechanical parameters: interaction forces. Kinematic parameters: (i) qualitative: average movement trajectories across participants; (ii) quantitative: movement duration, curvature, peak velocity, peak acceleration. EMG parameters: RMS of EMG normalized envelopes.	Participants were asked to rate statements concerning the perceived fatigue and comfort.	The analysis showed variations in EMG envelopes, longer movement durations, increased curvature, and reduced peak velocity and acceleration when wearing the device, along with higher EMG RMS values. Participants also reported decreased comfort and increased perceived difficulty while using the exoskeleton.
Souza et al. [36]	2024	Minimal interaction forces between the user and the device to avoid perturbing human motion.	Upper limb	Mechanical parameters: interaction forces (RMS over the trial and maximum absolute value). Kinematic parameters: movement duration, maximum size of the overshoot, and movement smoothness.	Subjective ranking of the controllers tested.	Predictive controllers significantly reduced the interaction force compared to non-predictive controllers, without affecting the kinematics of human motion. However, the participants did not perceive a noticeable difference in transparency between the different conditions.
Dalla Gasperina et al. [37]	2023	Capability of the device to not apply any resistive forces in reaction to intentional movements of the user.	Upper limb	Mechanical parameters: interaction forces and torques (mean and peak absolute joint residual torques). Kinematic parameters: RMSE between desired and actual positions, movement smoothness.	Participants were asked to rate statements concerning the device’s comfort and lack of restrictions. Participants also declared whether they preferred one controller in terms of comfort and ease of control.	The proposed novel controller allowed participants to perform precise and smooth movements with low interaction joint torques. Participants rated the new controller higher in comfort and agency, and lower in perceived resistance.
Stramel et al. [44]	2022	Capability of the device to not alter human natural movement.	Lower limb	Kinematic parameters: step length, stride time, step width. EMG parameters: peak EMG envelopes.	-	No significant effects were found on the user’s gait or muscle activation, indicating a good level of transparency.
Verdel et al. [42]	2022	Capability of the device to apply null interaction forces to minimally affect human movement both in terms of trajectories and in terms of muscular synergies and muscle activities.	Upper limb	Mechanical parameters: interaction efforts. Kinematic parameters: (i) qualitative: velocity profile; (ii) quantitative: peak velocity, peak acceleration, relative time to peak deceleration. EMG parameters: RMS of envelope, relationship between EMG and maximum acceleration (EMG/Acc index).	Participants had to answer questions regarding the perceived comfort, ability to move, and perceived movement precision achieved with the device.	Increasing the interaction area between the user and the device and adding passive degrees of freedom lead to improved interaction quality, also in terms of transparency.
Verdel et al. [33]	2021	Capability of the device to not change or influence human movement.	Upper limb	Kinematic parameters: movement duration, maximum velocity, isochrony principle (relationship between amplitude and duration or velocity). EMG parameters: relationship between EMG and maximum agonist acceleration (EMG/Acc index).	-	Although movements were performed significantly slower, the three tested control laws could be considered transparent, as they did not alter the bell-shaped velocity profile and preserved the isochrony principle. The FC control law showed greater performance in terms of the EMG/Acc index.
Camardella et al. [38]	2021	Capability of the device to not impede movements, ideally not being perceptible by the user.	Lower limb	Mechanical parameters: interaction forces. Kinematic parameters: gait smoothness (cross-correlation between joint angles), stride length.	-	The proposed novel control scheme showed increased transparency with respect to the compared ones, leading to reduced interaction forces, increased stride length, and a good level of gait smoothness.
Chiavenna et al. [52]	2018	Absence of interference in the user’s motor learning process, which is reflected in the preservation of motor modules due to the interaction with a device.	Upper limb	Synergy-based parameters: mean spatial synergy similarity, weight support features.	-	Muscular patterns were not significantly altered, whereas muscle activity (i.e., temporal coefficients of muscle synergies) was slightly reduced when support was not provided and consistently reduced during weight compensation.
Just et al. [39]	2018	Capability of the device to not apply any assistance/resistance to free motion, so that the robot’s reaction forces perceived by the user are minimal.	Upper limb	Mechanical parameters: interaction forces and torques.	Participants were asked to rate statements regarding perceived disturbances and difficulties when using the device.	The proposed novel controller improved the level of transparency with respect to the conventional one. Participants confirmed these results by reporting that the innovative controller leads to greater comfort and lower disturbance.
Bastide et al. [45]	2018	Control model that does not modify the nominal behavior of the user in terms of end-effector, joint trajectories, and patterns of muscle activations.	Upper limb	Mechanical parameters: absolute work, joint torque. Kinematic parameters: movement duration, mean velocity, relative time to peak velocity, isochrony principle, law of asymmetries.	-	Although the isochrony and asynchrony principles were conserved when wearing the device, movements became significantly slower, and the metabolic energy expenditure increased.
Jin et al. [46]	2017	Absence of additional perturbation from the device.	Lower limb	Kinematic parameters: step length, step height, knee maximum angle, knee RoM.	-	The RoMs and step length were significantly altered by the device, and compensating for the weight of the added mass only partially restored natural gait.
Fong et al. [49]	2017	Absence of unintentional forces that may compromise movement execution	Upper limb	Kinematic parameters: peak speed, time to peak speed, movement smoothness, curvature, movement accuracy.	-	The exoskeleton had an impact on the movement performance. However, the authors concluded that the alterations were significantly smaller with respect to other similar commercial devices.
Cai et al. [50]	2017	Capability of the device to not alter natural human movements and to minimally interfere with the user’s sensorimotor activity.	Lower limb	Mechanical parameters: zero moment point, joint torques. Kinematic parameters: joint angles.	-	The proposed gait phase detection method improved the device’s transparency.
Agarwal et al. [51]	2017	Absence of interference from the device in movement kinematics.	Thumb	Kinematic parameters: joint angles trajectory similarity.	-	The exoskeleton showed a good level of kinematic transparency: the nature of the movement was not altered, since the angle trajectories were preserved.
Pirondini et al. [43]	2016	Movement execution with and without the robot shows kinematically equivalent trajectories and similar patterns of muscle activation and coordination.	Upper limb	Kinematic parameters: mean distance, pace, number of peaks of the speed profile, differences in joint angular excursions across modalities. EMG parameters: RMS of EMG envelopes, spinal maps. Synergy-based parameters: muscle synergies, RMS of activation coefficients.	-	The use of the exoskeleton resulted in modifications of joint kinematics and alterations of muscle activities and motor control strategies. In particular, muscle activity levels were significantly reduced during assistance.
Fong et al. [47]	2015	Capability of the device to not apply any undesired or uncontrolled forces on the user’s limbs.	Upper limb	Kinematic parameters: peak speed, time to peak speed, movement smoothness, curvature, and accuracy.	-	The device significantly affected the reaching movements performed by the subjects: movements were, in general, slower and less smooth, indicating that the subjects found it more difficult to complete the task when wearing the exoskeleton.
Van Dijk et al. [41]	2013	Absence of undesired interaction forces between the device and the user.	Lower limb	Mechanical parameters: RMS of torque tracking error, RMS of interaction forces, RMS of interaction power.	-	The proposed novel controller improved torque tracking and reduced the interaction forces between the robot and the human, thereby improving the transparency of the robot.
Zanotto et al. [40]	2013	Capability of the device to have null interaction with the user when no corrective force is applied.	Lower limb	Mechanical parameters: interaction torques (RMS and mean interaction torques). Kinematic parameters: normalized RoM, mean joint angle, and stride length. EMG parameters: EMG envelope.	-	Wearing the device significantly affected RoMs, reduced cadence, and increased muscular effort. On the other hand, wearing only orthoses did not alter muscular activity.
Jarrassé et al. [5]	2010	Capability of the device to not resist intentional human motion, allowing natural, unperturbed movement	Upper limb	Mechanical parameters: interaction forces. Kinematic parameters: movement duration and smoothness, symmetry of velocity profile, trajectory curvature, final joint angles, RoM, cyclogram of shoulder–elbow angular velocity.	-	Movement duration and joint RoM increased. Moreover, the kinematic analysis demonstrated that subjects performed more corrections when interacting with the device, indicating that the robot leads to deviation from natural paths.

**Table 3:** Results of the sensitivity analysis.

Author	Year	Statistical Analysis	N of Citations (tot)	N of Citations Per Year	N of Subjects Included
Verdel et al. [35]	2024	Yes	9	9	14
Verdel et al. [33]	2021	Yes	33	8.3	6
Camardella et al. [38]	2021	Yes	29	7.3	11
Just et al. [39]	2018	Yes	42	6	20
Pirondini et al. [43]	2016	Yes	162	18	6

**Table 4:** Transparency assessment domains.

Transparency Assessment Domains	Metrics	Assessment Tools
Mechanical	Backdrivability, compliance, impendence, forces, torques, stiffness, weight distribution, inertia	F/T sensors, encoders
Kinematic	Position, velocity, acceleration, RoM, trajectory, movement quality	IMU, 3D cameras, marker-based systems
User perception	Subjective user perception (comfort, sense of freedom, usability, perceived functional effects…), motor control (muscle synergies)	Questionnaires, EMG sensors

## Data Availability

Not applicable.

## Abbreviations

The following abbreviations are used in this manuscript:

CNS	Central Nervous System
EMG	Electromyography
F/T	Force/Torque
IMU	Inertial Measurement Unit
MMF	Mixed Matrix Factorization
MSD	Musculoskeletal Disorder
MTC	Mean Temporal Component
PRISMA	Preferred Reporting Items for Systematic Reviews and Meta-Analyses
READ	Robot, Exoskeleton, and Assistive Device
RMS	Root Mean Square
RMSE	Root Mean Square Error
RoM	Range of Motion
RPE	Borg Rating of Perceived Exertion
SCI	Spinal Cord Injury
SEA	Series Elastic Actuator
SPARC	Spectral Arc Length
SUS	System Usability Scale
TAM	Technology Acceptance Model
TPV	Time to Peak Velocity
WRMSD	Work-Related Musculoskeletal Disorder
ZMP	Zero Moment Point

## References

[1] Bogue R. (2015). Robotic Exoskeletons: A Review of Recent Progress. Ind. Robot..

[2] Inoue Y., Kuroda Y., Yamanoi Y., Yabuki Y., Yokoi H. (2024). Development of Wrist Separated Exoskeleton Socket of Myoelectric Prosthesis Hand for Symbrachydactyly. Cyborg Bionic Syst..

[3] Wang Z., Xu D., Zhao S., Yu Z., Huang Y., Ruan L., Zhou Z., Wang Q. (2025). Level-Ground and Stair Adaptation for Hip Exoskeletons Based on Continuous Locomotion Mode Perception. Cyborg Bionic Syst..

[4] (2012). Robots and Robotic Devices–Vocabulary.

[5] Jarrassé N., Tagliabue M., Robertson J., Maiza A., Crocher V., Roby-Brami A., Morel G. (2010). A Methodology to Quantify Alterations in Human Upper Limb Movement during Co-Manipulation with an Exoskeleton. IEEE Trans. Neural Syst. Rehabil. Eng..

[6] Proietti T., Crocher V., Roby-Brami A., Jarrasse N. (2016). Upper-Limb Robotic Exoskeletons for Neurorehabilitation: A Review on Control Strategies. IEEE Rev. Biomed. Eng..

[7] Qiu S., Pei Z., Wang C., Tang Z. (2023). Systematic Review on Wearable Lower Extremity Robotic Exoskeletons for Assisted Locomotion. J. Bionic Eng..

[8] Xiloyannis M., Chiaradia D., Frisoli A., Masia L. (2019). Physiological and Kinematic Effects of a Soft Exosuit on Arm Movements. J. Neuroeng. Rehabil..

[9] de Looze M.P., Bosch T., Krause F., Stadler K.S., O’Sullivan L.W. (2016). Exoskeletons for Industrial Application and Their Potential Effects on Physical Work Load. Ergonomics.

[10] European Agency for Safety and Health at Work (EU-OSHA) Factsheet 71—Introduction to Work-Related Musculoskeletal Disorders 2013. https://osha.europa.eu/en.

[11] Publications Office of the European Union (2012). Eurofound Trends in Job Quality in Europe.

[12] Jakobsen L.S., Samani A., Desbrosses K., de Zee M., Steinhilber B., Madeleine P. (2025). Effects of 24-Weeks In-Field Use of a Back-Supporting Exoskeleton on Biomechanics, Work Intensity and Musculoskeletal Discomfort: A Randomized Controlled Trial Among Logistic Workers. Appl. Ergon..

[13] Moeller T., Krell-Roesch J., Woll A., Stein T. (2022). Effects of Upper-Limb Exoskeletons Designed for Use in the Working Environment—A Literature Review. Front. Robot. AI.

[14] Godwin A.A., Stevenson J.M., Agnew M.J., Twiddy A.L., Abdoli-Eramaki M., Lotz C.A. (2009). Testing the Efficacy of an Ergonomic Lifting Aid at Diminishing Muscular Fatigue in Women Over a Prolonged Period of Lifting. Int. J. Ind. Ergon..

[15] Feigin V.L., Brainin M., Norrving B., Martins S.O., Pandian J., Lindsay P., Grupper M.F., Rautalin I. (2025). World Stroke Organization: Global Stroke Fact Sheet 2025. Int. J. Stroke.

[16] Gassert R., Dietz V. (2018). Rehabilitation Robots for the Treatment of Sensorimotor Deficits: A Neurophysiological Perspective. J. Neuroeng. Rehabil..

[17] Wafa H.A., Wolfe C.D.A., Emmett E., Roth G.A., Johnson C.O., Wang Y. (2020). Burden of Stroke in Europe: Thirty-Year Projections of Incidence, Prevalence, Deaths, and Disability-Adjusted Life Years. Stroke.

[18] Cramer S.C. (2018). Treatments to Promote Neural Repair after Stroke. J. Stroke.

[19] Mehrholz J., Kugler J., Pohl M. (2012). Locomotor Training for Walking after Spinal Cord Injury. Cochrane Database Syst. Rev..

[20] Lanzani V., Brambilla C., Scano A. (2025). A Methodological Scoping Review on EMG Processing and Synergy-Based Results in Muscle Synergy Studies in Parkinson’s Disease. Front. Bioeng. Biotechnol..

[21] Williams K.L., Brauer S.G. (2022). Walking Impairment in Patients with Multiple Sclerosis: The Impact of Complex Motor and Non-Motor Symptoms across the Disability Spectrum. Aust. J. Gen. Pract..

[22] Peri E., Guanziroli E., Ferrante S., Pedrocchi A., Molteni F. (2018). Functional Electrical Stimulation and Its Use During Cycling for the Rehabilitation of Individuals with Stroke. Adv. Technol. Rehabil. Gait Balance Disord..

[23] Morone G., de Sire A., Martino Cinnera A., Paci M., Perrero L., Invernizzi M., Lippi L., Agostini M., Aprile I., Casanova E. (2021). Upper Limb Robotic Rehabilitation for Patients with Cervical Spinal Cord Injury: A Comprehensive Review. Brain Sci..

[24] Carpinella I., Cattaneo D., Abuarqub S., Ferrarin M. (2009). Robot-Based Rehabilitation of the Upper Limbs in Multiple Sclerosis: Feasibility and Preliminary Results. J. Rehabil. Med..

[25] Pirondini E., Coscia M., Marcheschi S., Roas G., Salsedo F., Frisoli A., Bergamasco M., Micera S. (2014). Evaluation of a New Exoskeleton for Upper Limb Post-Stroke Neuro-Rehabilitation: Preliminary Results. Replace, Repair, Restore, Relieve–Bridging Clinical and Engineering Solutions in Neurorehabilitation. Proceedings of the 2nd International Conference on NeuroRehabilitation (ICNR2014).

[26] Guidali M., Duschau-Wicke A., Broggi S., Klamroth-Marganska V., Nef T., Riener R. (2011). A Robotic System to Train Activities of Daily Living in a Virtual Environment. Med. Biol. Eng. Comput..

[27] Calabrò R.S., Sorrentino G., Cassio A., Mazzoli D., Andrenelli E., Bizzarini E., Campanini I., Carmignano S.M., Cerulli S., Chisari C. (2021). Robotic-Assisted Gait Rehabilitation Following Stroke: A Systematic Review of Current Guidelines and Practical Clinical Recommendations. Eur. J. Phys. Rehabil. Med..

[28] van Dijsseldonk R.B., van Nes I.J.W., Geurts A.C.H., Keijsers N.L.W. (2020). Exoskeleton Home and Community Use in People with Complete Spinal Cord Injury. Sci. Rep..

[29] Fritz H., Patzer D., Galen S.S. (2019). Robotic Exoskeletons for Reengaging in Everyday Activities: Promises, Pitfalls, and Opportunities. Disabil. Rehabil..

[30] Witte K.A., Fiers P., Sheets-Singer A.L., Collins S.H. (2020). Improving the Energy Economy of Human Running with Powered and Unpowered Ankle Exoskeleton Assistance. Sci. Robot..

[31] dos Santos L., Escalante F., Siqueira A.A.G., Boaventura T. IMU-Based Transparency Control of Exoskeletons Driven by Series Elastic Actuator. Proceedings of the IEEE 61st Conference on Decision and Control (CDC).

[32] Alonso V., de la Puente P. (2018). System Transparency in Shared Autonomy: A Mini Review. Front. Neurorobotics.

[33] Verdel D., Bastide S., Vignais N., Bruneau O., Berret B. (2021). An Identification-Based Method Improving the Transparency of a Robotic Upper Limb Exoskeleton. Robotica.

[34] Page M.J., McKenzie J.E., Bossuyt P.M., Boutron I., Hoffmann T.C., Mulrow C.D., Shamseer L., Tetzlaff J.M., Akl E.A., Brennan S.E. (2021). The PRISMA 2020 Statement: An Updated Guideline for Reporting Systematic Reviews. BMJ.

[35] Verdel D., Farr A., Devienne T., Vignais N., Berret B., Bruneau O. (2024). Human Movement Modifications Induced by Different Levels of Transparency of an Active Upper Limb Exoskeleton. Front. Robot. AI.

[36] Oliveira Souza A., Grenier J., Charpillet F., Ivaldi S., Maurice P. Enhancing Exoskeleton Transparency with Motion Prediction: An Experimental Study. Proceedings of the 2024 IEEE-RAS 23rd International Conference on Humanoid Robots (Humanoids).

[37] Dalla Gasperina S., Ratschat A.L., Marchal-Crespo L. Quantitative and Qualitative Evaluation of Exoskeleton Transparency Controllers for Upper-Limb Neurorehabilitation. Proceedings of the IEEE 2023 International Conference on Rehabilitation Robotics (ICORR).

[38] Camardella C., Porcini F., Filippeschi A., Marcheschi S., Solazzi M., Frisoli A. (2021). Gait Phases Blended Control for Enhancing Transparency on Lower-Limb Exoskeletons. IEEE Robot. Autom. Lett..

[39] Just F., Özen Ö., Bösch P., Bobrovsky H., Klamroth-Marganska V., Riener R., Rauter G. (2018). Exoskeleton Transparency: Feed-Forward Compensation vs. Disturbance Observer. Automatisierungstechnik.

[40] Zanotto D., Lenzi T., Stegall P., Agrawal S.K. Improving Transparency of Powered Exoskeletons Using Force/Torque Sensors on the Supporting Cuffs. Proceedings of the 2013 IEEE 13th International Conference on Rehabilitation Robotics (ICORR 2013).

[41] van Dijk W., van der Kooij H., Koopman B., van Asseldonk E.H.F., van der Kooij H. Improving the Transparency of a Rehabilitation Robot by Exploiting the Cyclic Behaviour of Walking. Proceedings of the 2013 IEEE 13th International Conference on Rehabilitation Robotics (ICORR 2013).

[42] Verdel D., Sahm G., Bastide S., Bruneau O., Berret B., Vignais N. (2022). Influence of the Physical Interface on the Quality of Human–Exoskeleton Interaction. IEEE Trans. Hum.-Mach. Syst..

[43] Pirondini E., Coscia M., Marcheschi S., Roas G., Salsedo F., Frisoli A., Bergamasco M., Micera S. (2016). Evaluation of the Effects of the Arm Light Exoskeleton on Movement Execution and Muscle Activities: A Pilot Study on Healthy Subjects. J. Neuroeng. Rehabil..

[44] Stramel D., Agrawal S. Assessing Changes in Human Gait with a Mobile Tethered Pelvic Assist Device (mTPAD) in Transparent Mode with Hand Holding Conditions. Proceedings of the 2022 9th IEEE RAS/EMBS International Conference for Biomedical Robotics and Biomechatronics (BioRob).

[45] Bastide S., Vignais N., Geffard F., Berret B. Interacting with a “Transparent” Upper-Limb Exoskeleton: A Human Motor Control Approach. Proceedings of the 2018 IEEE/RSJ International Conference on Intelligent Robots and Systems (IROS).

[46] Jin X., Cai Y., Prado A., Agrawal S. Effects of Exoskeleton Weight and Inertia on Human Walking. Proceedings of the 2017 IEEE International Conference on Robotics and Automation (ICRA).

[47] Fong J., Crocher V., Oetomo D., Tan Y. An Investigation into the Reliability of Upper-Limb Robotic Exoskeleton Measurements for Clinical Evaluation in Neurorehabilitation. Proceedings of the 2015 7th International IEEE/EMBS Conference on Neural Engineering (NER).

[48] Nurse C.A., Wolf D.N., Rodzak K.M., Teater R.H., Ice C.C., Fine S.J., Holtzman E.C., Zelik K.E. (2025). Evaluating the Biomechanical Effects and Real-World Usability of a Novel Ankle Exo for Runners. J. Biomech. Eng..

[49] Fong J., Crocher V., Tan Y., Oetomo D., Mareels I. EMU: A Transparent 3D Robotic Manipulandum for Upper-Limb Rehabilitation. Proceedings of the 2017 International Conference on Rehabilitation Robotics (ICORR).

[50] Cai V.A.D., Ibanez A., Nguyen T., Tam N. (2017). Transparency Enhancement for an Active Knee Orthosis by a Constraint-Free Mechanical Design and a Gait Phase Detection Based Predictive Control. Meccanica.

[51] Agarwal P., Yun Y., Fox J., Madden K., Deshpande A. (2017). Design, Control, and Testing of a Thumb Exoskeleton with Series Elastic Actuation. Int. J. Robot. Res..

[52] Chiavenna A., Scano A., Malosio M., Molinari Tosatti L., Molteni F. (2018). Assessing User Transparency with Muscle Synergies during Exoskeleton-Assisted Movements: A Pilot Study on the LIGHTarm Device for Neurorehabilitation. Appl. Bionics Biomech..

[53] Viviani P., Terzuolo C. (1982). Trajectory Determines Movement Dynamics. Neuroscience.

[54] Hensel R., Keil M. (2019). Subjective Evaluation of a Passive Industrial Exoskeleton for Lower-Back Support: A Field Study in the Automotive Sector. IISE Trans. Occup. Ergon. Hum. Factors.

[55] Hoffmann N., Prokop G., Weidner R. (2022). Methodologies for Evaluating Exoskeletons with Industrial Applications. Ergonomics.

[56] Golabchi A., Chao A., Tavakoli M. (2022). A Systematic Review of Industrial Exoskeletons for Injury Prevention: Efficacy Evaluation Metrics, Target Tasks, and Supported Body Postures. Sensors.

[57] Baldassarre A., Lulli L.G., Cavallo F., Fiorini L., Mariniello A., Mucci N., Arcangeli G. (2022). Industrial Exoskeletons from Bench to Field: Human-Machine Interface and User Experience in Occupational Settings and Tasks. Front. Public Health.

[58] Cardoso A., Ribeiro A., Carneiro P., Colim A. (2024). Evaluating Exoskeletons for WMSD Prevention: A Systematic Review of Applications and Ergonomic Approach in Occupational Settings. Int. J. Environ. Res. Public Health.

[59] Govaerts R., De Bock S., Provyn S., Vanderborght B., Roelands B., Meeusen R., De Pauw K. (2024). The Impact of an Active and Passive Industrial Back Exoskeleton on Functional Performance. Ergonomics.

[60] Rafique S., Rana S.M., Bjorsell N., Isaksson M. (2024). Evaluating the Advantages of Passive Exoskeletons and Recommendations for Design Improvements. J. Rehabil. Assist. Technol. Eng..

[61] Yu S., Huang T.-H., Yang X., Jiao C., Yang J., Chen Y., Yi J., Su H. (2020). Quasi-Direct Drive Actuation for a Lightweight Hip Exoskeleton With High Backdrivability and High Bandwidth. IEEE/ASME Trans. Mechatron..

[62] Liu C., Liang H., Ueda N., Li P., Fujimoto Y., Zhu C. (2020). Functional Evaluation of a Force Sensor-Controlled Upper-Limb Power-Assisted Exoskeleton with High Backdrivability. Sensors.

[63] Sanchez-Villamañan M.d.C., Gonzalez-Vargas J., Torricelli D., Moreno J.C., Pons J.L. (2019). Compliant Lower Limb Exoskeletons: A Comprehensive Review on Mechanical Design Principles. J. Neuroeng. Rehabil..

[64] Dalla Gasperina S., Longatelli V., Panzenbeck M., Luciani B., Morosini A., Piantoni A., Tropea P., Braghin F., Pedrocchi A., Gandolla M. AGREE: An Upper-Limb Robotic Platform for Personalized Rehabilitation, Concept and Clinical Study Design. Proceedings of the 2022 International Conference on Rehabilitation Robotics (ICORR).

[65] Lee K.H., Baek S.G., Lee H.J., Choi H.R., Moon H., Koo J.C. (2018). Enhanced Transparency for Physical Human-Robot Interaction Using Human Hand Impedance Compensation. IEEE/ASME Trans. Mechatron..

[66] Dalla Gasperina S., Roveda L., Pedrocchi A., Braghin F., Gandolla M. (2021). Review on Patient-Cooperative Control Strategies for Upper-Limb Rehabilitation Exoskeletons. Front. Robot. AI.

[67] Penna M.F., Giordano L., Tortora S., Astarita D., Amato L., Dell’Agnello F., Menegatti E., Gruppioni E., Vitiello N., Crea S. (2024). A Muscle Synergies-Based Controller to Drive a Powered Upper-Limb Exoskeleton in Reaching Tasks. Wearable Technol..

[68] Marchal-Crespo L., Reinkensmeyer D.J. (2009). Review of Control Strategies for Robotic Movement Training after Neurologic Injury. J. Neuroeng. Rehabil..

[69] Zhang T., Huang H. (2019). Design and Control of a Series Elastic Actuator With Clutch for Hip Exoskeleton for Precise Assistive Magnitude and Timing Control and Improved Mechanical Safety. IEEE/ASME Trans. Mechatron..

[70] Qian Y., Han S., Wang Y., Yu H., Fu C. (2023). Toward Improving Actuation Transparency and Safety of a Hip Exoskeleton With a Novel Nonlinear Series Elastic Actuator. IEEE/ASME Trans. Mechatron..

[71] Majidi C. (2014). Soft Robotics: A Perspective—Current Trends and Prospects for the Future. Soft Robot..

[72] Schmidt R.A. (1975). A Schema Theory of Discrete Motor Skill Learning. Psychol. Rev..

[73] Morasso P., Casadio M., Giannoni P., Masia L., Sanguineti V., Squeri V., Vergaro E. Desirable Features of a “Humanoid” Robot-Therapist. Proceedings of the 2009 Annual International Conference of the IEEE Engineering in Medicine and Biology Society.

[74] Alia C., Spalletti C., Lai S., Panarese A., Lamola G., Bertolucci F., Vallone F., Di Garbo A., Chisari C., Micera S. (2017). Neuroplastic Changes Following Brain Ischemia and Their Contribution to Stroke Recovery: Novel Approaches in Neurorehabilitation. Front. Cell Neurosci..

[75] Gandolla M., Ward N.S., Molteni F., Guanziroli E., Ferrigno G., Pedrocchi A. (2016). The Neural Correlates of Long-Term Carryover Following Functional Electrical Stimulation for Stroke. Neural Plast..

[76] Coscia M., Cheung V.C.K., Tropea P., Koenig A., Monaco V., Bennis C., Micera S., Bonato P. (2014). The Effect of Arm Weight Support on Upper Limb Muscle Synergies during Reaching Movements. J. Neuroeng. Rehabil..

[77] Cancrini A., Baitelli P., Nicora M.L., Malosio M., Pedrocchi A., Scano A. (2022). The Effects of Robotic Assistance on Upper Limb Spatial Muscle Synergies in Healthy People during Planar Upper-Limb Training. PLoS ONE.

[78] Scano A., Chiavenna A., Malosio M., Molinari Tosatti L., Molteni F. (2018). Robotic Assistance for Upper Limbs May Induce Slight Changes in Motor Modules Compared With Free Movements in Stroke Survivors: A Cluster-Based Muscle Synergy Analysis. Front. Hum. Neurosci..

[79] Wang C., Zhang S., Hu J., Huang Z., Shi C. (2021). Upper-Limb Muscle Synergy Features in Human-Robot Interaction with Circle-Drawing Movements. Appl. Bionics Biomech..

[80] Borg G.A. (1982). Psychophysical Bases of Perceived Exertion. Med. Sci. Sports Exerc..

[81] Brooke J. (1996). SUS: A “Quick and Dirty” Usability Scale. Usability Eval. Ind..

[82] Davis F.D. (1989). Perceived Usefulness, Perceived Ease of Use, and User Acceptance of Information Technology. MIS Q..

[83] d’Avella A., Portone A., Fernandez L., Lacquaniti F. (2006). Control of Fast-Reaching Movements by Muscle Synergy Combinations. J. Neurosci..

[84] Bizzi E., Cheung V.C.K., d’Avella A., Saltiel P., Tresch M. (2008). Combining Modules for Movement. Brain Res. Rev..

[85] Zhao K., Zhang Z., Wen H., Liu B., Li J., D’aVella A., Scano A. (2023). Muscle Synergies for Evaluating Upper Limb in Clinical Applications: A Systematic Review. Heliyon.

[86] Cheung V.C.K., d’Avella A., Tresch M.C., Bizzi E. (2005). Central and Sensory Contributions to the Activation and Organization of Muscle Synergies during Natural Motor Behaviors. J. Neurosci..

[87] Ivanenko Y.P., Cappellini G., Dominici N., Poppele R.E., Lacquaniti F. (2005). Coordination of Locomotion with Voluntary Movements in Humans. J. Neurosci..

[88] Ivanenko Y.P., Poppele R.E., Lacquaniti F. (2004). Five Basic Muscle Activation Patterns Account for Muscle Activity during Human Locomotion. J. Physiol..

[89] d’Avella A., Tresch M. (2001). Modularity in the Motor System: Decomposition of Muscle Patterns as Combinations of Time-Varying Synergies. Proceedings of the Advances in Neural Information Processing Systems.

[90] Brambilla C., Atzori M., Müller H., d’Avella A., Scano A. (2023). Spatial and Temporal Muscle Synergies Provide a Dual Characterization of Low-Dimensional and Intermittent Control of Upper-Limb Movements. Neuroscience.

[91] Scano A., Mira R.M., d’Avella A. (2022). Mixed Matrix Factorization: A Novel Algorithm for the Extraction of Kinematic-Muscular Synergies. J. Neurophysiol..

